# Supplementary material for: Engineered allogeneic T cells decoupling T-cell-receptor and CD3 signalling enhance the antitumour activity of bispecific antibodies
Source: Nat Biomed Eng. 2024 Sep 25;8(12):1665–81. doi: 10.1038/s41551-024-01255-x (PMC11668682; doi:10.1038/s41551-024-01255-x)
Supplement: Supplementary file 1 — Supplementary figures. [file 41551_2024_1255_MOESM1_ESM.pdf]

nature biomedical engineering

Supplementary information

https://doi.org/10.1038/s41551-024-01255-x

Engineered allogeneic T cells decoupling T-cell-receptor and CD3 signalling enhance the antitumour activity of bispecific antibodies

In the format provided by the authors and unedited

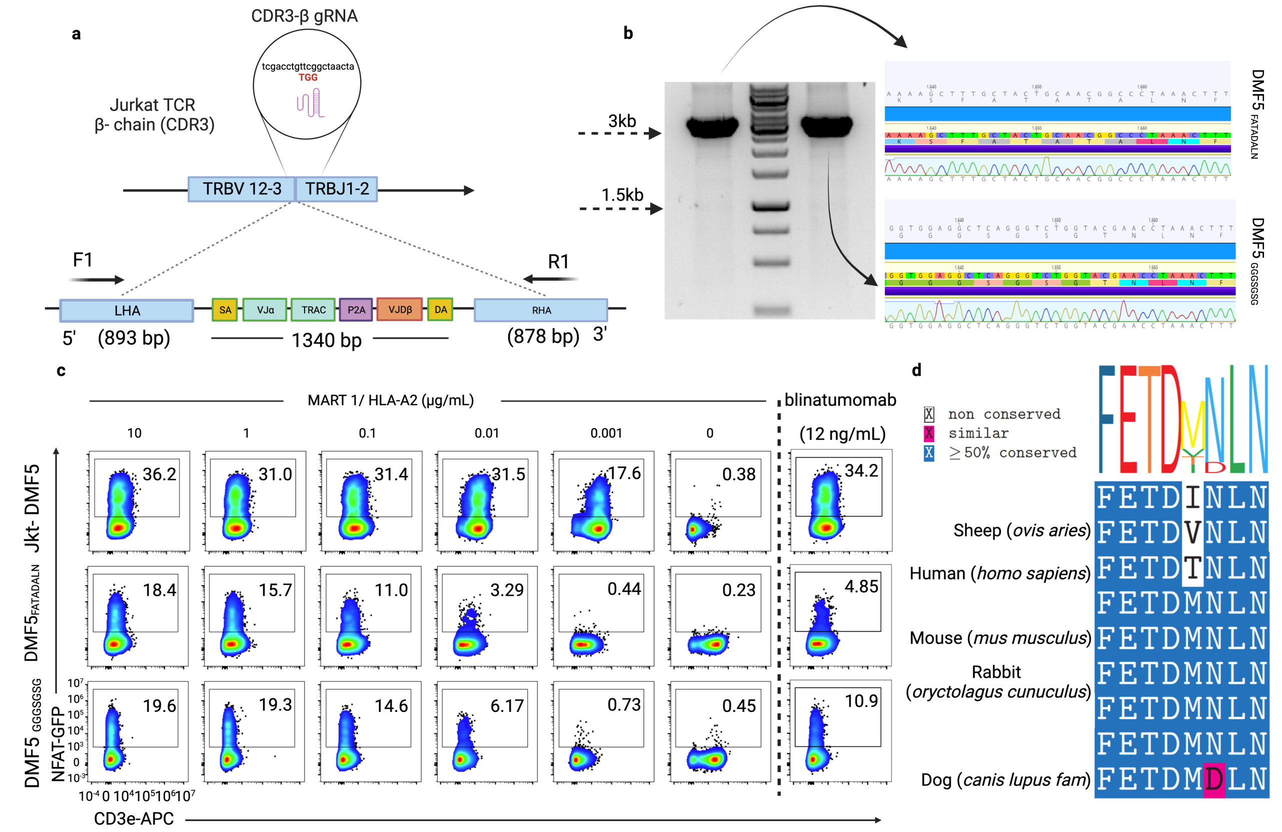

Supplementary Fig. 1 I Generation of FETDxNLN DMF5 variants and flow cytometry results. a. The CDR3 region of the Jurkat T-cell β chain is targeted with a specific sgRNA. LHA and RHA (Left and Right homology arms), SA (splice acceptor), complete α- DMF5 chain, P2A coding sequence, VJD-β-DMF5 chain containing the splice donor (SD) which splices with the endogenous TRBC. The entire homology-directed repair (HDR) template is 3111 bp long and is PCR-amplified with the F1 (GCATGGATCCCAATGC) and R1 (TTTTATCTGTCATGGCCGTGACCG) primers. **b.** Gel electrophoresis and sequencing confirm the integration of Jkt- DMF5 variants into the correct gDNA locus. **c.** Representative flow cytometry plots of the Jkt-DMF5 and its FETDxNLN variants in response to ELAGIGILTV peptide. The activation is measured by NFAT-GFP expression. **d.** Sequence alignment of the TRAC domain across species signifies the preserved FETDxNLN motif in the α- connecting peptide motif (aCP). Panel a was created with Biorender.com.

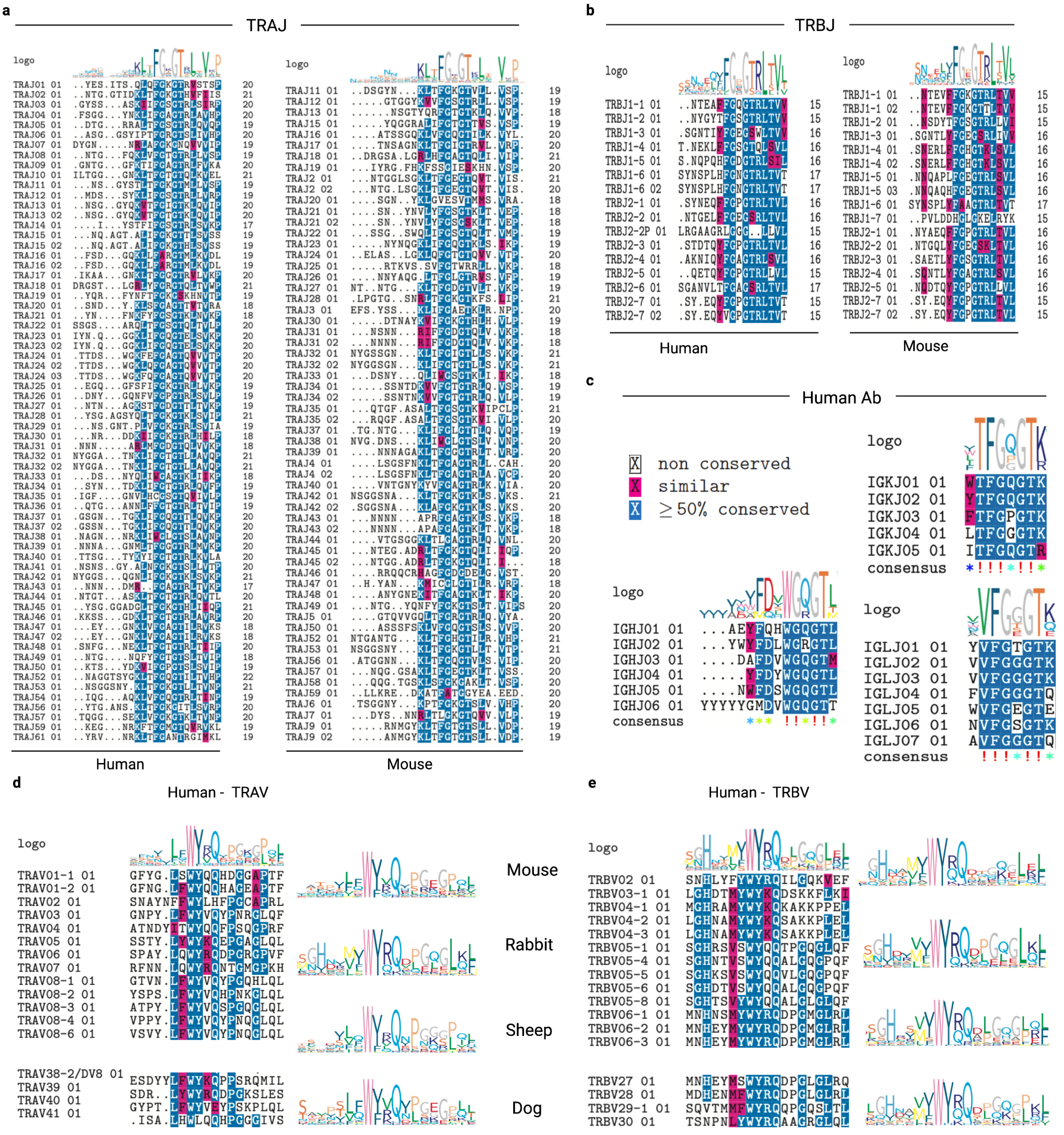

Supplementary Fig. 2 | Alignment of TCR and antibody germline sequences. a. List of TRAJ sequences in the human and murine genome and the conserved FGxGT motif. **b.** List of human and murine TRBJ sequences and the presence of the FGxGT motif. **c.** Human heavy and light chain antibody sequences depicting WGxGT and FGxGT motif. **d.** Sequence analysis of a set of selected TRAV regions and the position of the WYxQ motif in the human genome and additional listed species. **e.** Selected TRBV human regions and the WYxQ motif across listed species.

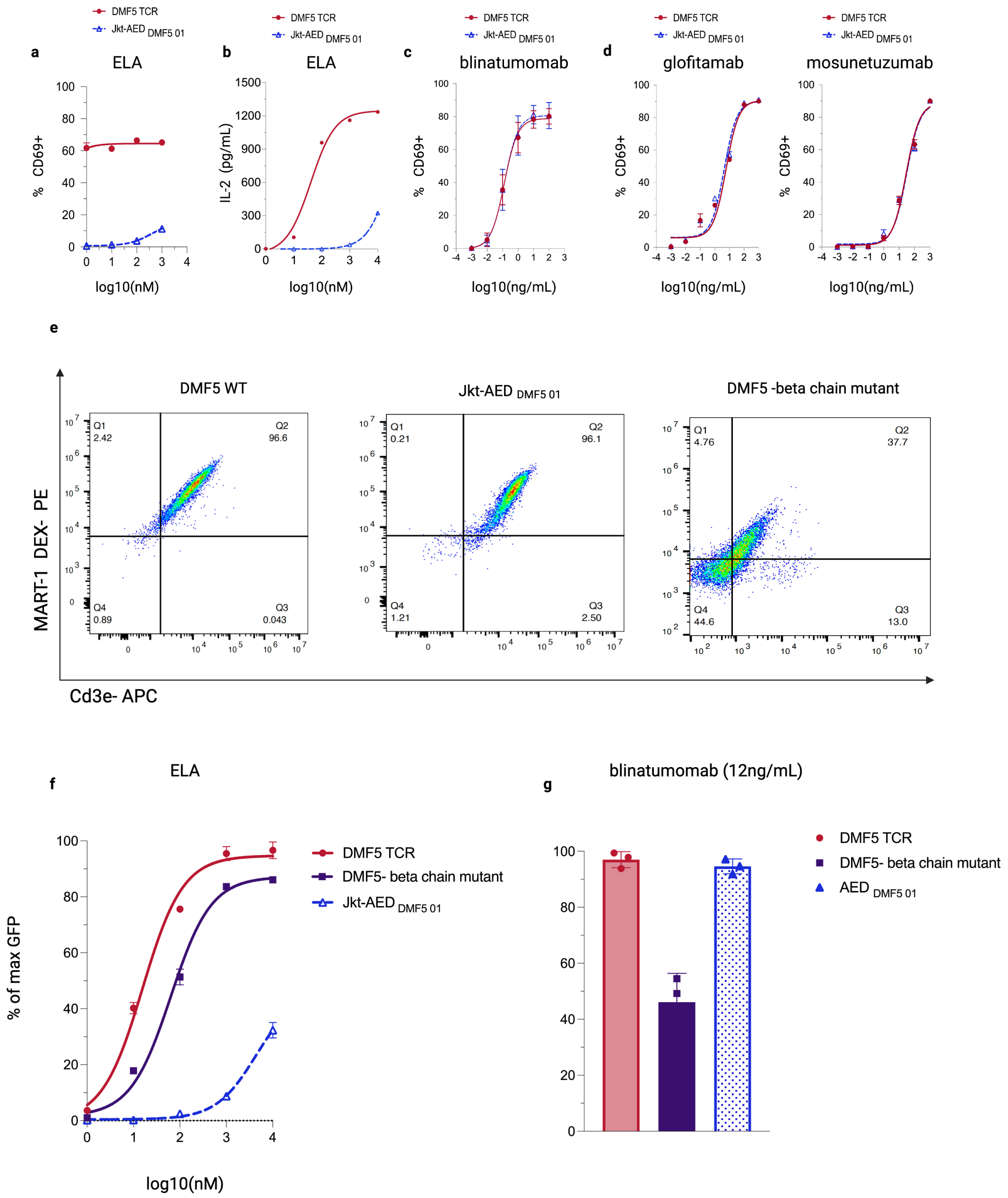

Supplementary Fig. 3 l TCR binding and activation to peptide/blinatumomab. a. Jkt-DMF5 TCR and Jkt-AED$_{DMF5\ 01}$ were tested for CD69+ expression with the ELA peptide. **b.** IL-2 concentration was measured in the supernatant after 24h co-culture with ELA-peptide pulsed T2 cells. **c.** CD69, early T activation marker, measured over extended blinatumomab concentration (0-100 ng/mL) **d.** CD69 expression of AED T cells compared to WT when activated with mosunetuzumab and glofitamab (0-1000ng/mL) **e.** MART-1 DEX and CD3 staining depict the differences in antibody binding between DMF5 WT, AED$_{DMF5\ 01,}$ and DMF5-beta chain mutant. **f.** GFP expression of the WT DMF5, Jkt-AED$_{DMF5\ 01,}$ and DMF5-beta chain mutant across stimulated with the ELA-peptide pulsed T2 cells. **g.** GFP expression of WT DMF5, Jkt-AED$_{DMF5\ 01,}$ and DMF5-beta chain mutant when stimulated with blinatumomab (12 ng/mL). Data are normalized to the maximum GFP expression. Symbols, medians of 3 technical replicates. Error bars, s.d. (**a,b,c,d,f,g**)

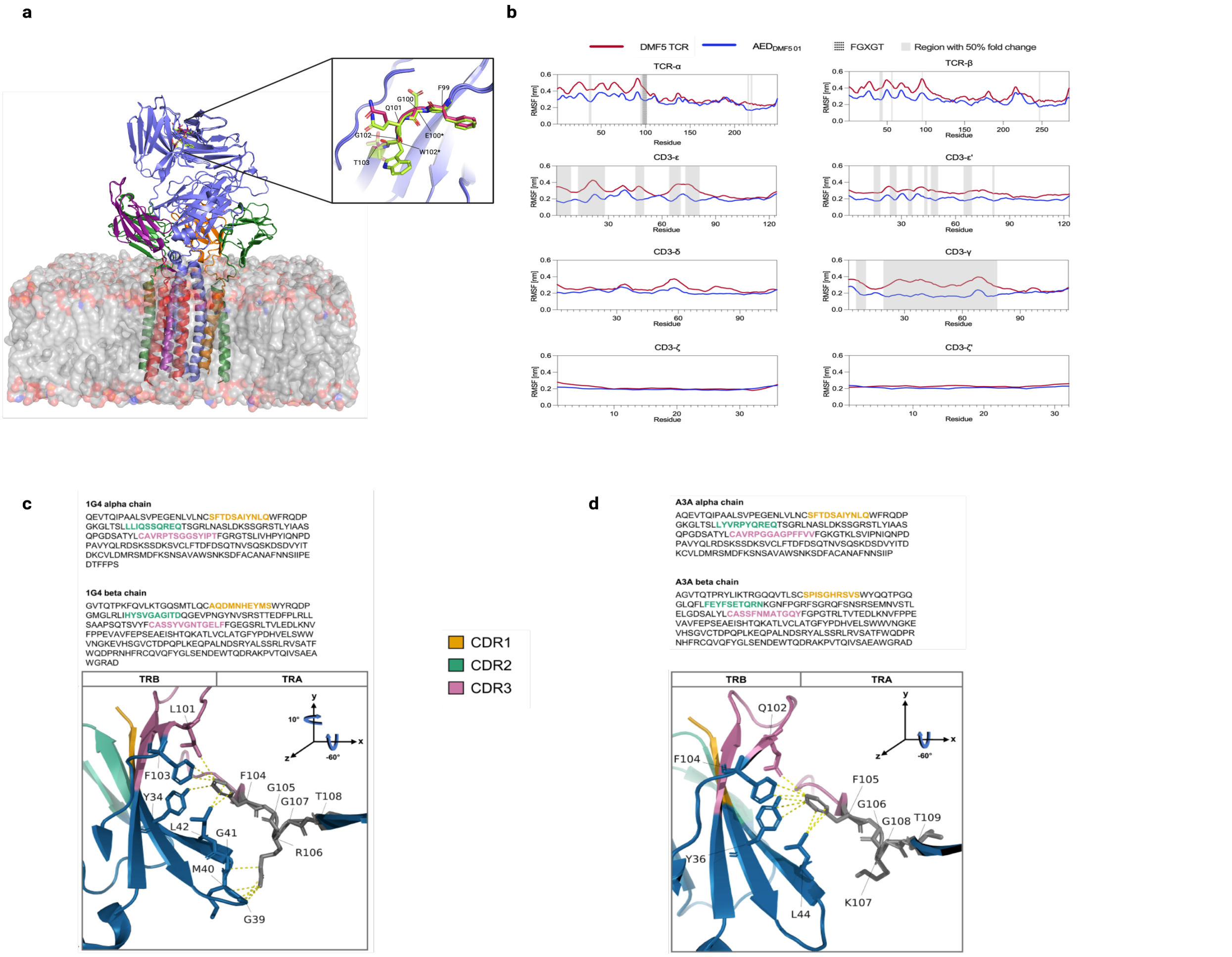

Supplementary Fig. 4 | Molecular dynamics of DMF5 TCR and AED$_{DMF501}$ and structural analysis of 1G4 and a3a TCRs. a. In silico molecular dynamics (MD) modeling and overlap of DMF5 TCR and AED$_{DMF501}$ embedded in the lipid bilayer with the CD3 molecules. Zoomed in segment depicts the overlay of the FGxGT (magenta) and FEQWT mutation (limon) **b.** RMSF flexibility changes between the DMF5 TCR and AED$_{DMF5\,01}$ were observed across the individual chains of the TCR/CD3 complex. Mutations introduced in the FGxGT region show the potential to affect the dynamics of different chains, potentially affecting the overall TCR functional characteristics. **c.** 1G4 TCR (PDB (2BNR) is specific for HLA A2*01 MHC and SLLMWITQC peptide (orange). Complementary determining regions (CDRs) are color-coded (CDR1-yellow, CDR-2-green, CDR3-magenta) and a complete TCR sequence (alpha and beta chain) is depicted in the upper right corner. On the right, a close-up of the FGXGT motif (in grey) and its interaction with the corresponding amino acids in the β-chain is shown. F104 (α- chain) forms multiple bonds with the adjacent amino acids in the β- chain. **d.** a3aTCR (PDB 5B3Z) is specific for HLA A01*01 and EVDPIGHLY peptide (orange). The panel consists of the same elements as panel c and the close-up highlights the F105 conserved interactions with the a3a β-chain TCR (PDB 5B3Z) is specific for HLA A01*01 and EVDPIGHLY peptide (orange). The panel consists of the same elements as panel **c** and the close-up highlights the F105 conserved interactions with the a3a β-chain.

a

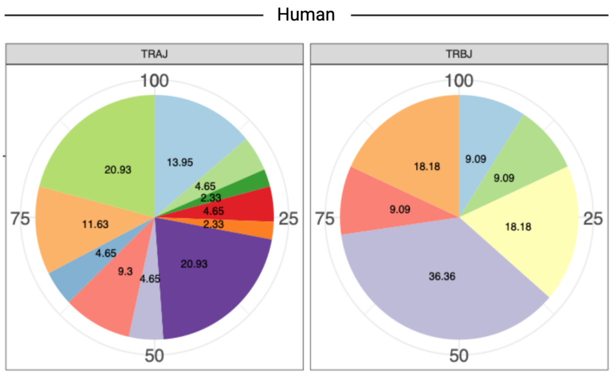

b

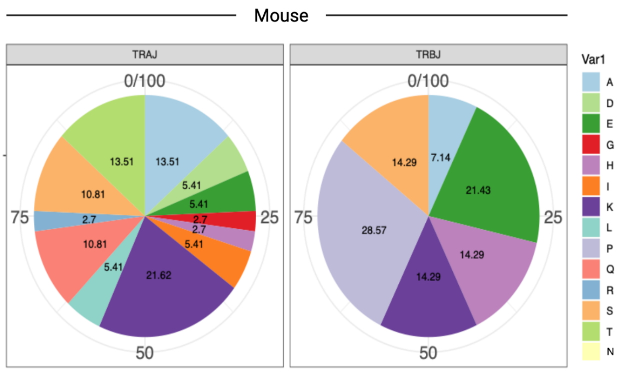

c

MHC	TCR	Target proteins	peptide(s)	TRAV	TRAJ	TRBV	TRBJ	TRBD	PDB	CDR3a
HLA-A*02:01	DMF5	MART-1	AAGIGILTV, EAGIGILTV, ELAGIGILTV (synthetic)	12-2*01	23*01	6-4*01	1-1*01	2*01	3QDJ	AVNFGGGKLI
HLA-A*02:01	1G4	NY-ESO-1	SLLMWITQC	21*01	6*01	6-5*01	2-2*01	2*01	2BNR	AVRPTSGGSYIPT
HLA-A*01:01	A3A	MAGE- A3	EVDPIGHLY	21*01	28*01	5-1*01	2-7*01	2*01	5BRZ	AVRPGGAGPFFVV

d

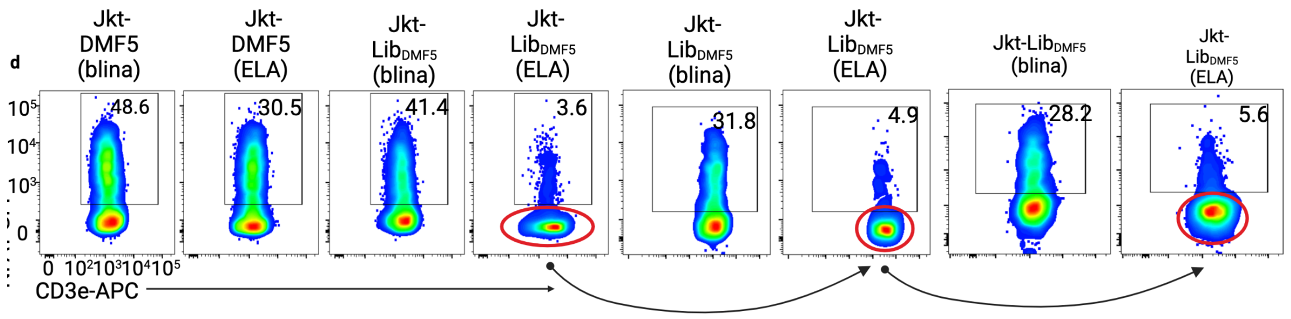

Supplementary Fig. 5 | FGxGT variance across J-genes and TCR library FACS selection. a and **b** the “x” amino acid in the FGxGT motif is more diverse in TRAJ than in TRBJ germline sequences. Lysine (K) and Threonine (T) are the most prevalent amino acids in human TRAJ domains, while in mouse TRAJ Lysine (K) is the most frequent. **c.** Table depicting the TCRs used in the library screening, the α- and β- chain composition and HLA- peptide specificity. **d.** Representative flow cytometry plots of library selection rounds. Peptide negative fraction was selected as a starting Jkt- population for the following round. Each TCR library was enriched 3 times. A peptide concentration of 100 ng/mL was used as a threshold to separate high- and low-response T cell populations. Blinatumomab was used at a concentration of 12 ng/mL.

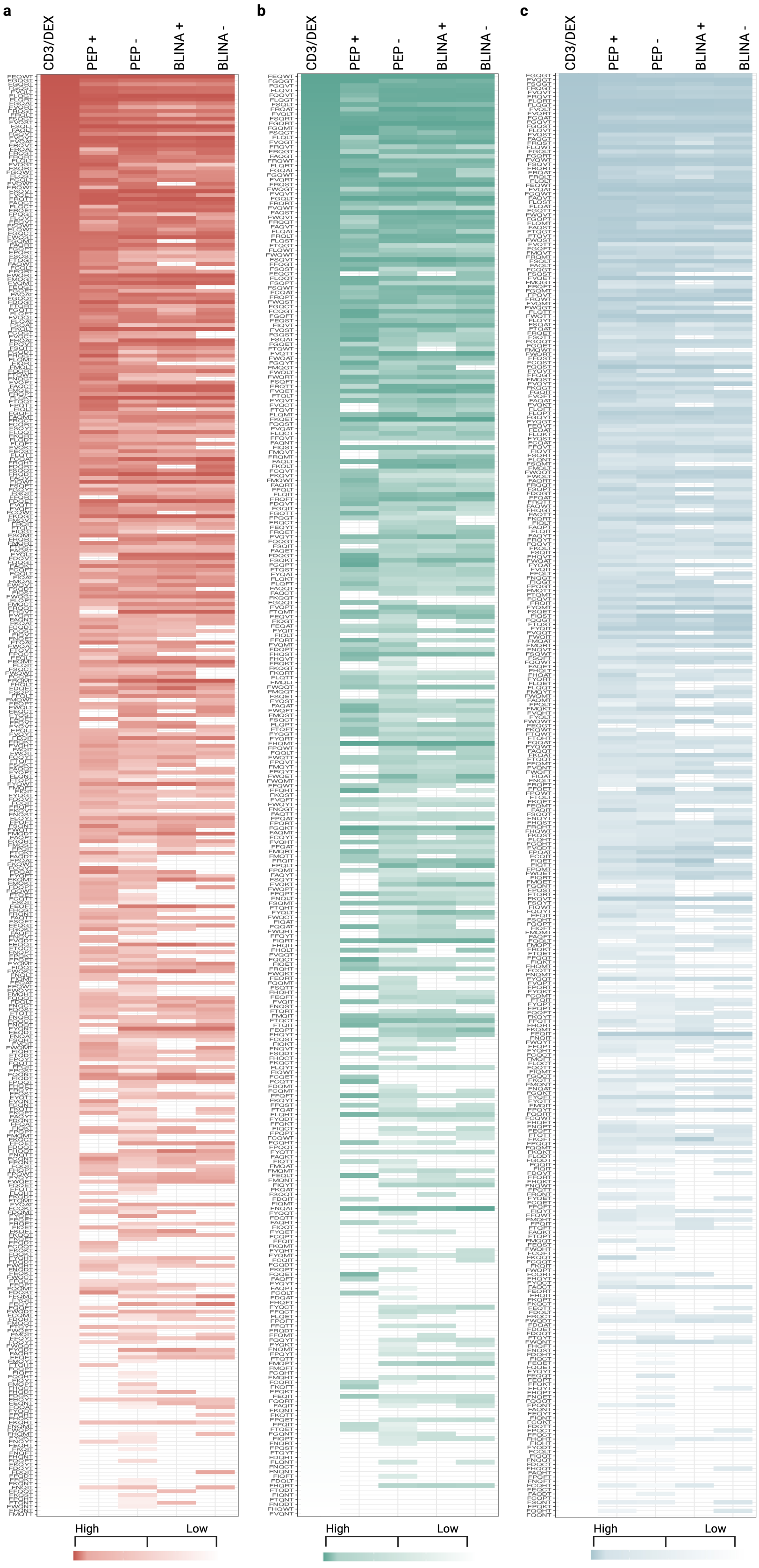

d

	TCR name	FGxGT variant
1	DMF5	FGQGT
2	AED$_{DMF5\ 01}$	FEQWT
3	AED$_{DMF5\ 02}$	FWQST
4	AED$_{DMF5\ 03}$	FHQAT
5	AED$_{DMF5\ 04}$	FYQLT
6	AED$_{DMF5\ 05}$	FHQVT

e

	TCR name	FGxGT variant
1	1G4	FGKGT
2	AED$_{1G4\ 01}$	FSQVT
3	AED$_{1G4\ 02}$	FVQLT
4	AED$_{1G4\ 03}$	FLQGT
5	AED$_{1G4\ 04}$	FRQAT
6	AED$_{1G4\ 05}$	FKQET

f

	TCR name	FGxGT variant
1	A3A	FGKGT
2	AED$_{A3A\ 01}$	FWQST
3	AED$_{A3A\ 02}$	FVQLT
4	AED$_{A3A\ 03}$	FIQST

g

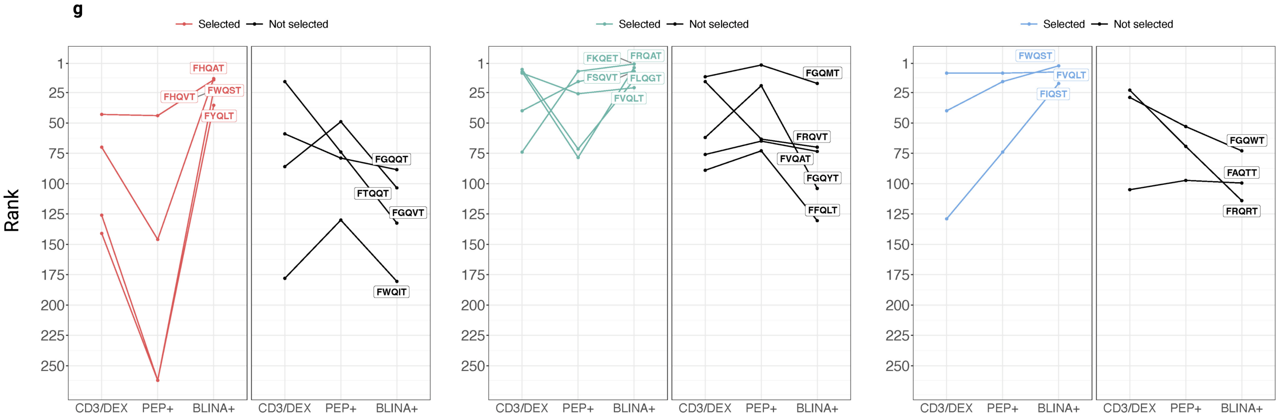

h

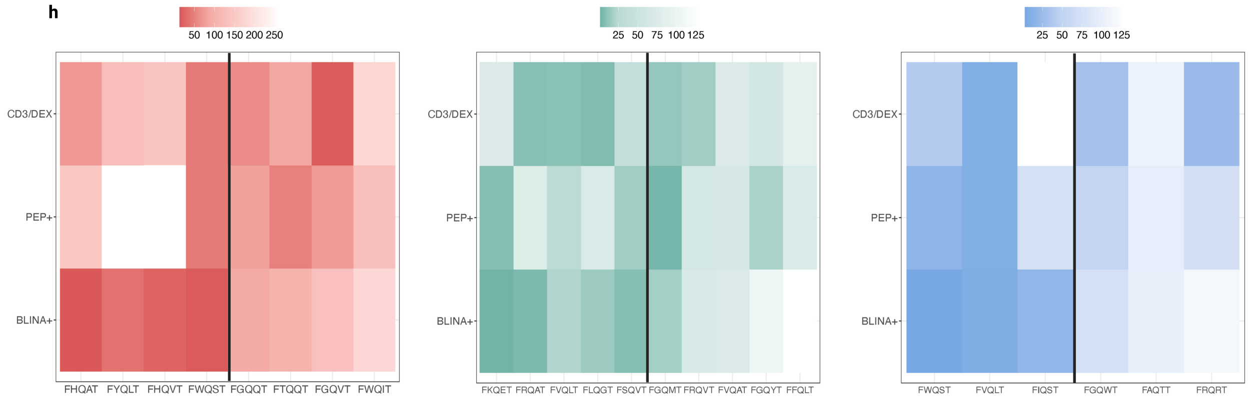

Supplementary Fig. 6 | TCR library enrichment heat maps and selection of AED variants. a-c. Heat maps of DMF5 (red), 1G4 (green), and a3a (blue) TCR library variants. The color of the tile indicates the rank at which the variant was observed. White tiles indicate missing observations. **d-f.** FGxGT sequences of selected TCR variants from each library. **g**. Change in the rank of selected variants in CD3/DEX+, PEP+, and BLINA+ populations. A group of not selected variants is also represented. **h.** Heat maps of DMF5 (red), 1G4 (green), and a3a (blue) TCR library variants. A heat map representation of selected AED variants (left of the black line) and several TCR variants that are not fulfilling one or more criteria (right of the black line). An appropriate AED candidate must be present in the CD3/DEX+ group, underrepresented in the peptide+ (PEP+) group, and overrepresented in the blinatumomab+ group (BLINA+). The color of the tile indicates the rank at which the variant was observed. White tiles indicate missing observations.

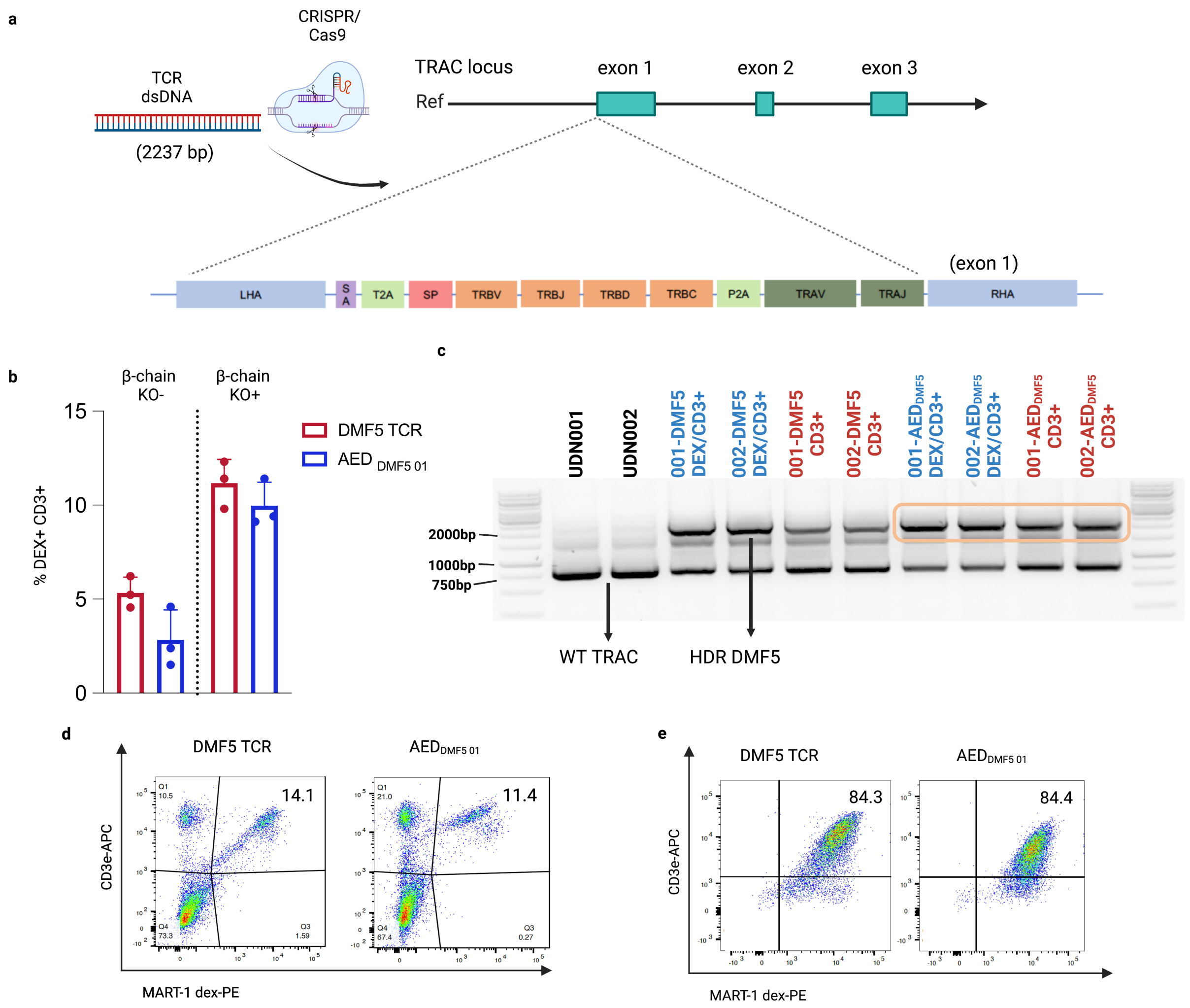

Supplementary Fig. 7 l Primary Human T cells engineering and transfection efficiency. **a.** Cas9- mediated integration was performed with the sgRNA targeting the exon1 of the TRAC locus. The complete DMF5 TCR was introduced in frame with the endogenous TRAC. **b.** The bar plot shows the difference in DMF5 TCR and $AED_{DMF5\ 01}$ surface expression without and with simultaneous β-chain knock-out (KO). **c.** Sequencing of genomic DNA (gDNA) of CD3+ and CD3/dex+ cells show genomic integration even in the CD3+ only fraction. The orange rectangle represents the even split in AED integration between the CD3+ and CD3+/DEX+ integration. **d-e.** DMF5 TCR and $AED_{DMF5\ 01}$ T transfection efficiency (b) and sorted purity (c) prior to the co-culture experiment. Symbols, medians of 3 biological replicates. Error bars, s.d. (**b**)Panel a was created with Biorender.com.

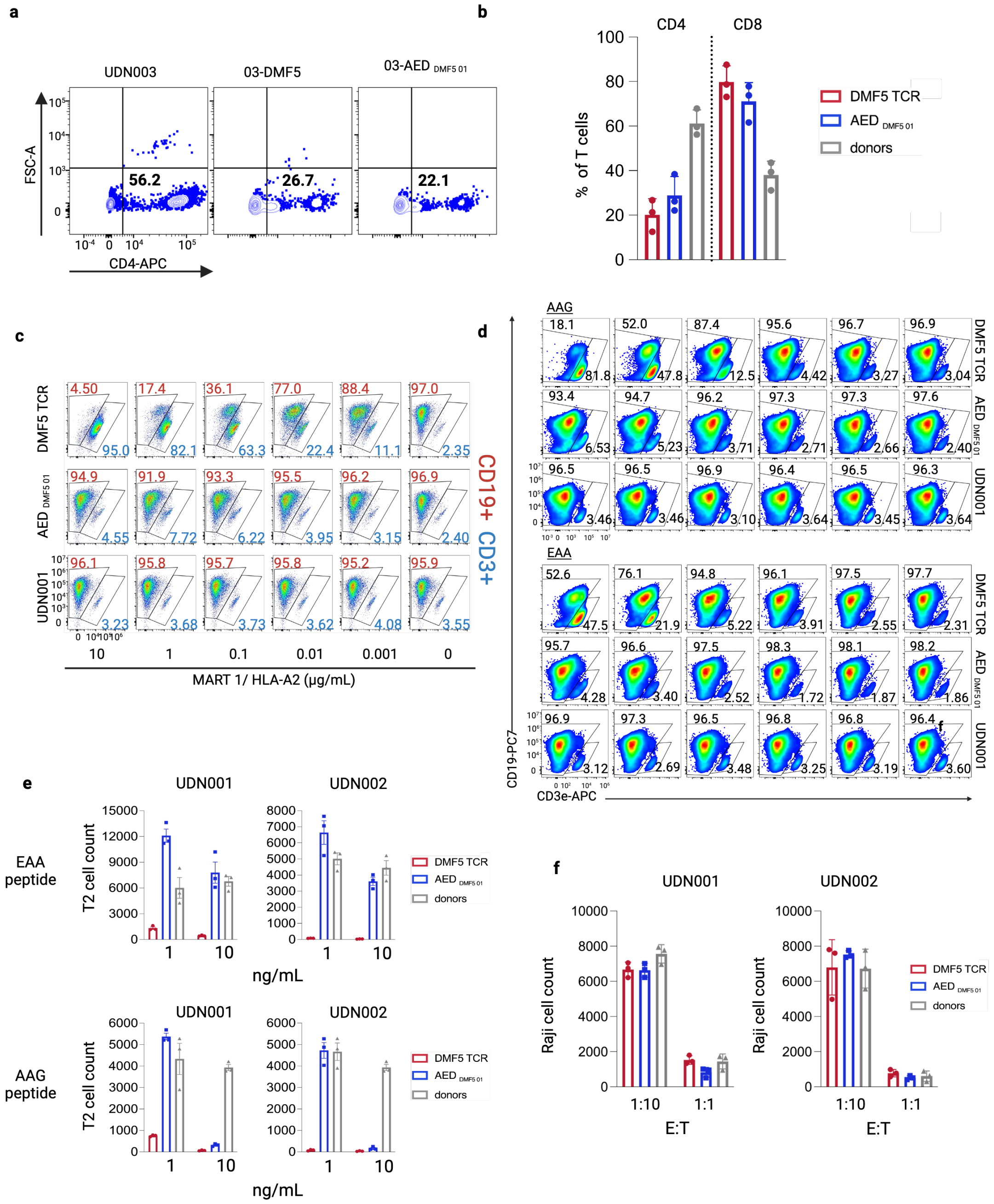

Supplementary Fig. 8 | Primary T cells *in vitro* MART-1 peptide and blinatumomab assays.
a. Representative flow cytometry plots to display the uneven distribution of CD4+ T -cells between the donors and genetically engineered DMF5 T cells **b.** The bar plot shows the distribution of CD4+ and CD8+ T cells across donors and engineered variants (DMF5 TCR and $AED_{DMF5\ 01}$). Symbols, medians of 3 biological replicates. Error bars, s.d.. **c-d.** Representative flow cytometry plots for the T-cell proliferation in co-culture with ELA, AAG, and EAA peptide-pulsed T2 cells. UDN001 donor is shown. **e.** Peptide-pulsed T2 cells for physiological peptide expression (1 and 10 ng/mL). Symbols, medians of 3 technical replicates, 2 donors. Error bars, s.d. , 1:1 T cells to T2 cell ratio **f.** Raji cell counts induced in the presence of blinatumomab. Symbols, medians of 3 technical replicates, 2 donors. Error bars, s.d. , 1:10 and 1:1 T cells to target cells ratio.

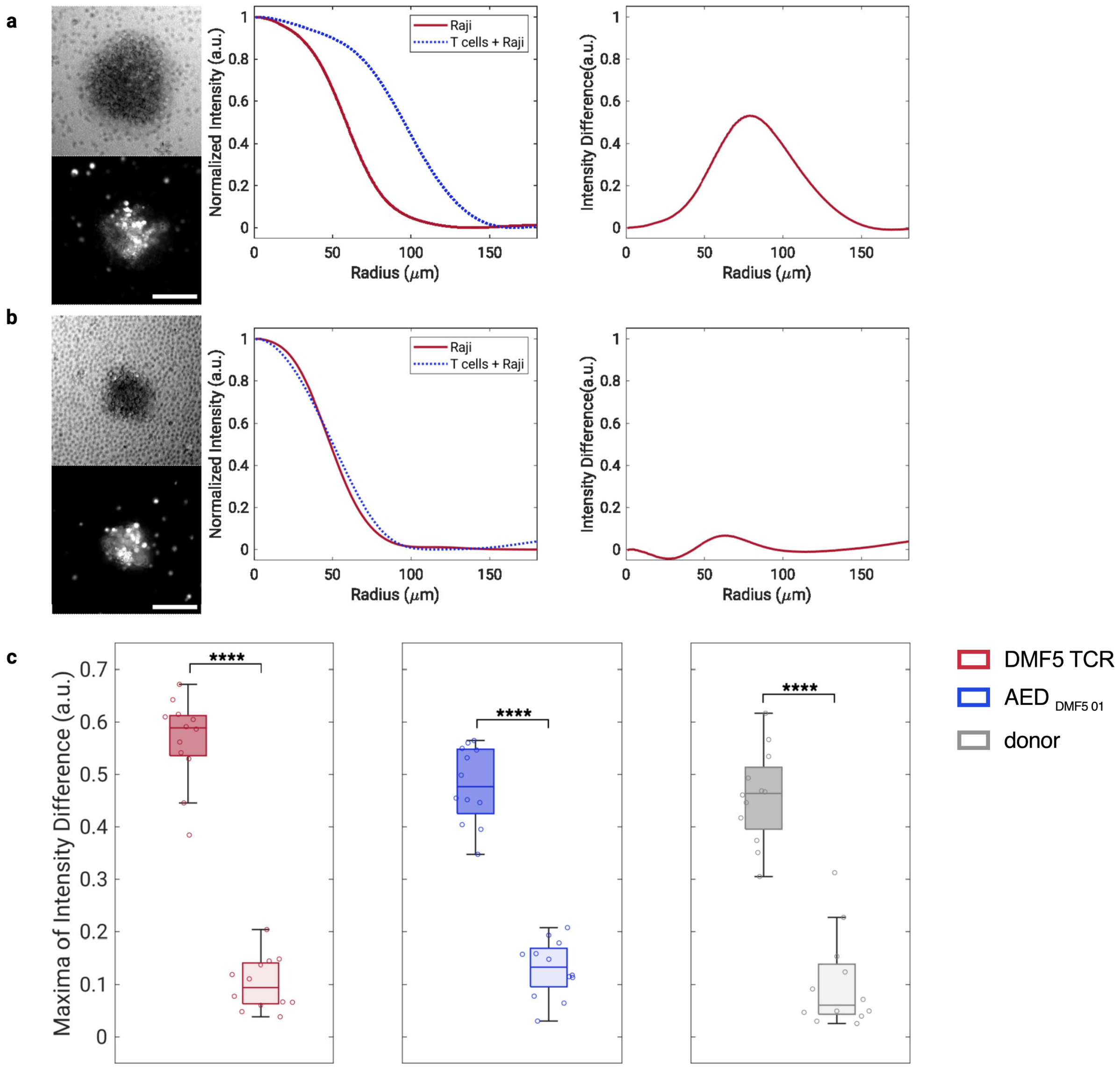

Supplementary Fig. 9 l Image analysis of the Raji/T cell co-culture. a. Microscopy image representing an example cluster of T cells + Raji cells (bright field image, top) and Raji cells (fluorescence image, bottom) with (**a**) and without blinatumomab (**b**). The scale bar's width is 100 μm. The radial intensity of the clusters is normalized in the fluorescent images (Raji, red) and bright-field images (T cells+ Raji, red) **c.** The maxima of normalized intensity difference for each T cell group with and without blinatumomab is plotted (n= 12 clusters). The line in the box represents the median value and whiskers minimum and maximum values measured. the P values were determined with one-way ANOVA with Tukey's correction for multiple comparisons * P < 0.05,** P < 0.01, *** P < 0.001, **** P < 0.0001, ns = not significant. Significant P values >0.001 are numerically written.

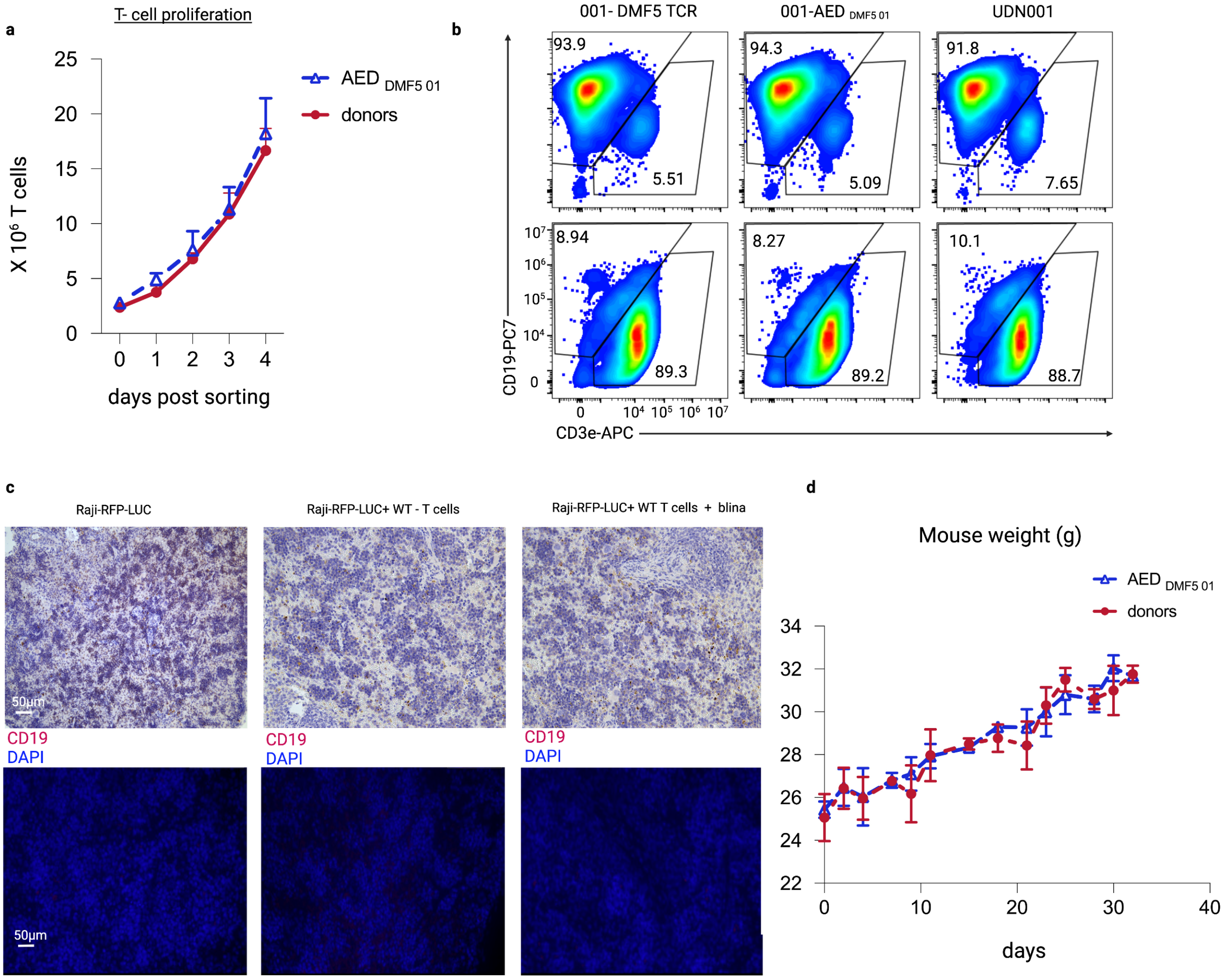

Supplementary Fig. 10 I *In vitro* blinatumomab response and mouse spleen microscopy imaging. a. post-sorting proliferation of T cells used in mouse experiments. **b.** T-cell proliferation in co-culture with Raji cells with and without the addition of blinatumomab. No unspecific alloresponse was observed in the samples with UDN001 cells without blinatumomab. The same pattern was observed for other two donors (data not shown). **c.** Chromogenic staining of spleens shows no presence of CD3+ T cells, only background staining is observed. Immunohistochemistry staining with CD19 (red) and DAPI (blue) is not showing any presence of Raji (CD19+) cells in the spleen. **d.** Mouse weight monitoring curves depict no differences in mouse weight between mouse groups receiving $AED_{DMF5\ 01}$ or donor T cells. Symbols, means of 2 donors (**a**) or 5 mice(**d**). Error bars, s.d.
