# Supplementary material for: Engineered allogeneic T cells decoupling T-cell-receptor and CD3 signalling enhance the antitumour activity of bispecific antibodies
Source: Nat Biomed Eng. 2024 Sep 25;8(12):1665–81. doi: 10.1038/s41551-024-01255-x (PMC11668682; doi:10.1038/s41551-024-01255-x)
Supplement: Supplementary file 2 — Reporting Summary [file 41551_2024_1255_MOESM2_ESM.pdf]

nature portfolio

Corresponding author(s): Sai T. Reddy

Last updated by author(s): Jun 21, 2024

Reporting Summary

Nature Portfolio wishes to improve the reproducibility of the work that we publish. This form provides structure for consistency and transparency in reporting. For further information on Nature Portfolio policies, see our Editorial Policies and the Editorial Policy Checklist.

Statistics

For all statistical analyses, confirm that the following items are present in the figure legend, table legend, main text, or Methods section.

n/a	Confirmed	
☐	☒	The exact sample size (*n*) for each experimental group/condition, given as a discrete number and unit of measurement
☐	☒	A statement on whether measurements were taken from distinct samples or whether the same sample was measured repeatedly
☐	☒	The statistical test(s) used AND whether they are one- or two-sided<br>*Only common tests should be described solely by name; describe more complex techniques in the Methods section.*
☒	☐	A description of all covariates tested
☐	☒	A description of any assumptions or corrections, such as tests of normality and adjustment for multiple comparisons
☐	☒	A full description of the statistical parameters including central tendency (e.g. means) or other basic estimates (e.g. regression coefficient) AND variation (e.g. standard deviation) or associated estimates of uncertainty (e.g. confidence intervals)
☒	☐	For null hypothesis testing, the test statistic (e.g. *F*, *t*, *r*) with confidence intervals, effect sizes, degrees of freedom and *P* value noted<br>*Give P values as exact values whenever suitable.*
☒	☐	For Bayesian analysis, information on the choice of priors and Markov chain Monte Carlo settings
☒	☐	For hierarchical and complex designs, identification of the appropriate level for tests and full reporting of outcomes
☒	☐	Estimates of effect sizes (e.g. Cohen's *d*, Pearson's *r*), indicating how they were calculated

Our web collection on statistics for biologists contains articles on many of the points above.

Software and code

Policy information about availability of computer code

Data collection	IVIS Lumina II imaging system for in vivo mouse imaging. LSRFortessa (BD Biosciences) or a CytoFLEX(Beckman- Coulter) were used to collect flow-cytometry data. Cells were sorted using BD FACSAria III or BD FACSAria Fusion instruments. Leica DM5500 Upright Microscope and Nikon Ti2 were used for microscopy imaging.
Data analysis	FlowJo 10, Geneious 11.1.4, Microsoft Excel for Mac, GraphPad Prism 9, QuPath 02.03, R (version 4.0.1.), MATLAB.

For manuscripts utilizing custom algorithms or software that are central to the research but not yet described in published literature, software must be made available to editors and reviewers. We strongly encourage code deposition in a community repository (e.g. GitHub). See the Nature Portfolio guidelines for submitting code & software for further information.

Data

Policy information about availability of data

All manuscripts must include a data availability statement. This statement should provide the following information, where applicable:

- Accession codes, unique identifiers, or web links for publicly available datasets
- A description of any restrictions on data availability
- For clinical datasets or third party data, please ensure that the statement adheres to our policy

All data generated in this study, including source data for the figures, are available as Source data or from the corresponding author on reasonable request.

Research involving human participants, their data, or biological material

Policy information about studies with human participants or human data. See also policy information about sex, gender (identity/presentation), and sexual orientation and race, ethnicity and racism.

Reporting on sex and gender	The study did not involve human participants.
Reporting on race, ethnicity, or other socially relevant groupings	–
Population characteristics	–
Recruitment	–
Ethics oversight	–

Note that full information on the approval of the study protocol must also be provided in the manuscript.

Field-specific reporting

Please select the one below that is the best fit for your research. If you are not sure, read the appropriate sections before making your selection.

☒ Life sciences ☐ Behavioural & social sciences ☐ Ecological, evolutionary & environmental sciences

For a reference copy of the document with all sections, see nature.com/documents/nr-reporting-summary-flat.pdf

Life sciences study design

All studies must disclose on these points even when the disclosure is negative.

Sample size	Statistical tests to predetermine sample size were not performed. We used 5 mice per experimental group (in the Raji-alone group we used 3 mice) to observe statistically meaningful differences between groups.
Data exclusions	No data were excluded from analysis.
Replication	The experimental results were replicated with several healthy human donors, and all attempts at replication were successful.
Randomization	For the in vivo experiments, mice were randomly distributed and injected with tumor cells and T cells. All experimental groups contained equal proportions of male and female animals. All mice were imaged on the same day of AED T/tumor cell injection, and tumor burden was consistent and reproducible across all mice.
Blinding	The researchers were not blinded, to ensure appropriate data acquisition and handling.

Reporting for specific materials, systems and methods

We require information from authors about some types of materials, experimental systems and methods used in many studies. Here, indicate whether each material, system or method listed is relevant to your study. If you are not sure if a list item applies to your research, read the appropriate section before selecting a response.

Materials & experimental systems

n/a	Involved in the study	
☐	☒	Antibodies
☐	☒	Eukaryotic cell lines
☒	☐	Palaeontology and archaeology
☐	☒	Animals and other organisms
☒	☐	Clinical data
☒	☐	Dual use research of concern
☒	☐	Plants

Methods

n/a	Involved in the study	
☒	☐	ChIP-seq
☐	☒	Flow cytometry
☒	☐	MRI-based neuroimaging

Antibodies

Antibodies used	The following antibodies were used in this study. From Biolegend: APC-CD3e(clone UCHT1 #300458) PE-Cy7- CD3e (clone UCHT1, #300420), APC- CD4 (clone RPA-T4, #300552), PE-CD8a (clone HIT8a, #300908), PE-Cy7-CD19 (clone HIB19, #302216), PE-conjugated

anti-human TCR α/β (clone IP26, #306707);PE anti-human CD107a (LAMP-1)antibody (clone H4A3, #328608); DAPI viability dye (Thermo Fisher, #62248);. Sytox Blue viability dye (Invitrogen, # S34857);CountBright Plus counting beads (Invitrogen, # C36995) .The following peptide-MHC dextramers were commercially obtained from Immudex: NY-ESO-1157-165 (SLLMWITQC, HLA-A*0201, #WB2696-PE); MART-126-35(27L) (ELAGIGILTV, HLA-A*0201, #WB2162-PE); MAGE-A3168-176 (EVDPIGHLY, HLA-A*0101, #WA3249-PE).

Validation	All antibodies were validated by the respective manufacturer.

Eukaryotic cell lines

Policy information about cell lines and Sex and Gender in Research

Cell line source(s)	Cell lines were originally obtained from ATCC.
Authentication	The cell lines were not authenticated beyond the ATCC provided certificates of analysis. Cell morphology and surface expression of antigens were consistent with published data.
Mycoplasma contamination	All cell lines were routinely tested for mycoplasma, and the results were negative.
Commonly misidentified lines (See ICLAC register)	No commonly misidentified cell lines were used.

Animals and other research organisms

Policy information about studies involving animals; ARRIVE guidelines recommended for reporting animal research, and Sex and Gender in Research

Laboratory animals	NSG (NOD.Cg-PrkdcSCIDIl2rgtm1Wjl/Sz) breeder pair mice were purchased from Charles River. Mice were bred in the EPFL mouse facility in accordance with IACUC regulations. Both female and male litter mates (5 weeks of age) were used for the experiments.
Wild animals	The study did not involve wild animals.
Reporting on sex	Both female and male litter mates (aged 5 weeks) were used in the experiments.
Field-collected samples	The study did not involve samples collected from the field.
Ethics oversight	All experiments were performed according to protocols approved by the EPFL IACUC.

Note that full information on the approval of the study protocol must also be provided in the manuscript.

Flow Cytometry

Plots

Confirm that:

- [x] The axis labels state the marker and fluorochrome used (e.g. CD4-FITC).
- [x] The axis scales are clearly visible. Include numbers along axes only for bottom left plot of group (a 'group' is an analysis of identical markers).
- [x] All plots are contour plots with outliers or pseudocolor plots.
- [x] A numerical value for number of cells or percentage (with statistics) is provided.

Methodology

Sample preparation	For flow cytometry and cell sorting, cells were washed in (PBS 2% FBS) and incubated 20 minutes at 4 deg in FACS buffer (PBS 2% FBS, 2 mM EDTA) containing the desired antibodies.
Instrument	Cell sorting (FACS) was performed using BD FACSAria III or BD FACSAria Fusion instruments.
Software	FlowJo 10 software (BD Biosciences).
Cell population abundance	Flow cytometry was employed to ensure the purity of a target cell populations.
Gating strategy	FSC/SSC gating was used to define an initial lymphocyte gate together with the singlet gate.

- [x] Tick this box to confirm that a figure exemplifying the gating strategy is provided in the Supplementary Information.
